# Retraction Note: Culturomics and Amplicon-based Metagenomic Approaches for the Study of Fungal Population in Human Gut Microbiota

**DOI:** 10.1038/s41598-024-58471-3

**Published:** 2024-04-02

**Authors:** Ibrahim Hamad, Stéphane Ranque, Esam I. Azhar, Muhammad Yasir, Asif A. Jiman-Fatani, Hervé Tissot-Dupont, Didier Raoult, Fadi Bittar

**Affiliations:** 1 Aix Marseille University, AP-HM, URMITE, IHU Méditerranée Infection, IRD 198, Inserm 1095, 7278 CNRSMarseille, France; 2 Charmo Research Center, Charmo University, 46023 Chamchamal, Sulaimani Iraq; 3 Special Infectious Agents Unit, King Fahd Medical Research Centre, King Abdulaziz University, Jeddah, Saudi Arabia; 4 Department of Medical Laboratory Technology, Faculty of Applied Medical Sciences, King Abdulaziz University, Jeddah, Saudi Arabia; 5 Department of Medical Microbiology and Parasitology, Faculty of Medicine, King Abdulaziz University, Jeddah, Saudi Arabia

Retraction of: *Scientific Reports* 10.1038/s41598-017-17132-4, published online 01 December 2017

Editors have retracted this Article.

After publication of this paper concerns about ethical oversight of this study were brought to the attention of the Editors. The paper cites approval from an institutional ethics committee in France, but samples used in this study were also sourced from Algeria, Saudi Arabia, and Niger. The Authors were not able to provide documentation of approval from ethics committees in these countries, or of compliance with local regulations regarding the use of such samples in research.

Stéphane Ranque, Esam I. Azhar, Muhammad Yasir, Asif A. Jiman-Fatani, Didier Raoult, and Fadi Bittar disagree with the retraction. Ibrahim Hamad and Hervé Tissot-Dupont did not reply to the correspondence from the Editors.

